# Factors associated with status and self-perceived mental health changes in the face of the COVID-19 pandemic in Brazil

**DOI:** 10.1371/journal.pgph.0001636

**Published:** 2023-08-18

**Authors:** Rander Junior Rosa, Juliana Soares Tenório de Araújo, Thaís Zamboni Berra, Antônio Carlos Vieira Ramos, Heriederson Sávio Dias Moura, Murilo César do Nascimento, Ariela Fehr Tártaro, Ruan Victor dos Santos Silva, Felipe Mendes Delpino, Regina Célia Fiorati, Titilade Kehinde Ayandeyi Teibo, Yan Mathias Alves, Juliana Queiroz Rocha de Paiva, Marcos Augusto Moraes Arcoverde, Alessandro Rolim Scholze, Ricardo Alexandre Arcêncio

**Affiliations:** 1 Department of Maternal-Infant Nursing and Public Health, University of São Paulo at Ribeirão Preto College of Nursing, Ribeirão Preto, São Paulo, Brazil; 2 Department of Neurosciences and Behavioral Sciences, Ribeirão Preto Medical School of the University of São Paulo, Ribeirão Preto, São Paulo, Brazil; 3 Center for Education, Letters and Health, Western Paraná State University, Campus Foz do Iguaçu, Foz do Iguaçu, Paraná, Brazil; 4 Department of Nursing, State University of North Paraná, Bandeirantes, Paraná, Brazil; University of California Irvine, UNITED STATES

## Abstract

The SARS-CoV-2-triggered Public Health Emergency of International Importance has significantly contributed to emotional and mental health issues. The aim of this study was to identify factors associated with self-perceived mental health changes while facing the COVID-19 pandemic in Brazil. This was a cross-sectional, descriptive, and analytical study that collected data via a web survey using a validated instrument. The study included individuals over 18 years old residing in the 26 federal units and the Federal District from August 2020 to November 2022. The sample was recruited using the snowball technique. Two logistic regression analyses were conducted to identify factors associated with the outcomes of interest. The first analysis considered individuals who rated their mental health condition as poor as the dependent variable, while the second analysis considered individuals who reported changes in their mental health during the pandemic as the dependent variable. The study found that individuals with complete college education and those using tranquilizers or antidepressants were more likely to perceive their mental health as poor (1.97 and 2.04 times higher likelihood, respectively). Increased consumption of ultra-processed foods during the pandemic was associated with a 2.49 higher likelihood of reporting mental health changes. Participants also reported more difficulty sleeping. The negative self-perception of mental health varied across Brazil’s regions and changed over time, with different patterns observed before and after the vaccination period. In 2022, most regions of Brazil classified their mental health as "poor." The study highlights the impact of the COVID-19 pandemic on mental health, with increased prevalence of mental disorders and emotional problems among the population. The results highlight the presence of mental disorders and increased reporting of emotional problems among the population due to the impact of the COVID-19 pandemic.

## Introduction

The COVID-19 pandemic had immeasurable impacts on the lives and health of people around the world. Since 2020, the Pan American Health Organization (PAHO) has warned about the increase in mental suffering caused by the emergence of the new coronavirus. This is due to the strategies used for its containment, such as physical isolation, as the various containment measures put in place have affected the mental wellbeing of the population, causing an increase in psychological suffering and psychic symptoms [1]. In addition, we can highlight other causes of mental suffering related to the pandemic, such as fear of high risk of infection, deaths of close people and COVID-19 itself, in addition to the failure of policies to protect the population. In this sense, the pandemic had a great impact on the mental health of the population, which led to an increase in the prevalence of depression, anxiety disorders, suicide risk, post-traumatic stress symptoms and insomnia [2].

The psychological repercussions of a pandemic must be considered and observed. Fear of infection, deaths, and the disease itself expressed high levels of concern for people’s mental health during the COVID-19 pandemic. The fear of being infected by COVID-19 has direct consequences on the daily lives and mental health of populations. The number of people with psychological problems tends to be greater than the number of people affected by the COVID-19 infection. Fear of infection increases levels of anxiety, distress, lack of sleep and frustration in healthy individuals and intensifies pre-existing psychiatric symptoms [3].

In France, there were differences in mental health trajectories during the pandemic, as people with elevated symptoms experienced worsened mental health during periods of restrictive measures, which improved after the restrictions ended. On the other hand, people with low initial mental health symptoms also showed deterioration over time, but did not return to the initial mental health level during the COVID-19 pandemic [4]. Already among Americans with a history of depression, for example, the pandemic negatively implied on routines, access to mental health treatment, alcohol use, use of prescription painkillers, and use of other drugs [5].

The increase in mental symptoms and mental disorders during the pandemic occurred for several reasons. Among them, one can highlight the direct action of the COVID-19 virus on the central nervous system [6]. Other possible reasons include; traumatic experiences associated with the infection or the death of close relatives, stress induced by change in routine due to social distancing measures or by its economic consequences, work routine, disruption of affective relationships, interruption of treatment due to access difficulties, among others. People exposed to more than one of the mentioned conditions may have an increased risk to develop or to aggravate already existing mental disorders [7].

In 2003, during the severe acute respiratory syndrome (SARS) epidemic, a study showed that people consulted three times more with psychiatrists than with infectologists one year after infection. This demonstrates the importance of post-infection mental health care, as the decrease in psychological well-being can significantly contribute to the emergence of mental disorders, as well as to the evolution of its clinical picture in the general population, in addition, when not correctly treated and followed-up there is increase in the chances of the individual progressing to death/suicide and self-mutilation [8].

The onset of mental disorders depends on complex mechanisms such as neuro functional factors, exposure to environmental stressors, and biological susceptibility (epigenetics). It is worth noting that environmental factors can alter gene expression, so, exposure to extremely unfavorable environmental conditions associated with increased social vulnerability, further exacerbated by the COVID-19 pandemic, can trigger mental disorders even in individuals without genetic predisposition, and this may explain the epidemic potential for post-pandemic mental health changes [9].

The hidden epidemic of mental disorders has extremely worrying potential for society, both from an individual and collective health perspective. While it is still all very new, the combined effect of these factors is already beginning to be evidenced through studies addressing the risk of mental disorders because of coronavirus infections [10].

It is posited that the Public Health Emergency of International Importance initiated with the spread of SARS-CoV-2 has greatly affected people’s mental health. An in-depth analysis of scientific literature on the impact of the initial stages of the COVID-19 pandemic on Latin American psychology is alarming. Studies showed that when quarantine planning began, a set of psychosocial and communication psychology strategies that could have made some measures more effective in mitigating the negative impacts on the well-being and health of the population were not in place [11].

Although there is a dense body of published work on this topic in the literature, more research on mental health needs to specify and explain which factors are associated with self-perception of their mental health. In this way, it is necessary to advance knowledge about mental health and factors associated with it in the face of the health crisis experienced in Brazil. In this context, the objective of this study was to identify factors associated with self-care and perceived changes in mental health while coping with the COVID-19 pandemic in Brazil.

## Materials and methods

### Study design and site

This is a cross-sectional, descriptive and analytical study [12], with data collection via web survey. The study covered 26 units of the federation and the Brazilian Federal District, in the period between August 2020 to November 2022.

### Study population and sample definition

The study population was made up of people who declared that they were Brazilians or foreign migrants who understood the language spoken in Brazil (Brazilian Portuguese) and had lived in Brazil for at least six months, 18 years of age or older, and who were willing to participate in the survey- having internet access available.

The sample was enrolled using the snowball technique [13], a non-probabilistic technique in which participants were invited to respond to counseling through the websites of institutions participating in the research, e-mail, WhatsApp, social media (Facebook, Instagram, Twitter), in addition to inviting people from the contact network of the investigators involved [14].

It should be noted that this type of initiative was chosen given the scenario of the research carried out in Brazil, a country with continental dimensions composed of 5,568 municipalities, and this type of approach makes it easier to find audiences in addition to being a low-cost procedure. cost and easy to carry out, since the members of the sample themselves indicate other members and already help in the initial contact for carrying out the research.

It is worth noting that the snowball technique does not require calculation of margin of error and confidence level because it is a non-probabilistic method. From the above, the calculation for finite populations was used, using the formula below [15].

$$N=\frac{\frac{ZZ^{2}X_{P}(1-p)}{e^{2}}}{1+(\frac{Z^{2}X(1-p)}{e^{2}N})}$$

where N = population size; margin of error of 5% and p = standard deviation of 50%, losses of 10%, reaching a minimum sample of 1428 participants.

### Instrument and data collection

The instrument used in the first stage was based on one adapted from questions existing in the National Health Survey of Portugal [16]. Together with the COVID-19 Rapid Quantitative Assessment Tool of WHO [17], validated and published in distinct studies [18–20] of a group of researchers from the National School of Public Health of the New University of Lisbon (ENSP-UNL). For application in Brazil, there was cultural adaptation and validation by researchers from the National School of Public Health at Fiocruz (ENSP-FIOCRUZ) and Ribeirão Preto School of Nursing at the University of São Paulo (EERP-USP), through the Delphi technique and was called "COVID-19 Social Thermometer: Social Opinion".

All aesthetics of the instrument and also its availability was done through the software REDCap [21]. Participants were invited to answer the questionnaire by accessing the link which was widely disseminated through the websites of the institutions participating in the research using e-mail, WhatsApp, social networks (Facebook, Instagram, Twitter) or blogs, in addition, people were invited from the network of contacts of the researchers involved. In addition, the participants were instructed to recruit other people from their social circle to participate in the survey, in order to obtain the expected sample.

### Data analysis

After consistent analysis of the database, exploratory analyses were performed to characterize the profile of the people who answered the questionnaire.

### Logistic regression

Regression was used based on the variables present in the instrument used in the study, namely: Age (18 to 39 years, 40 to 59); Sex (Female and Male), Race/color (black/brown and white), Religion (no and yes), Receives government aid (no and yes), Left home during the pandemic (no and yes), Belongs to the group of workers with greater exposure to COVID-19 (no and yes), Occupation/position (formal work, informal work, student or no job), Monthly income (less than one minimum wage, 1 to 5 minimum wages, 5 to 10 minimum wages, above 10 minimum wages), Education (no education, complete high school, complete elementary school, higher education or postgraduate), Felt calm or cheerful after the isolation measures (no and yes). During the pandemic what have you done to cope with the situation (increased consumption of ultra-processed foods, started smoking or increased the amount of cigarettes, increased consumption of alcoholic beverages, started or increased the practice of physical activities and/or relaxation techniques), Compared to the period before COVID-19, how you have been feeling (felt less agitated, anxious or tense, felt less irritable, felt less sad, less discouraged or cried less easily, felt less overwhelmed, can now do all tasks, no problems sleeping, no changes in your feelings) Because of COVID-19, started or increased the use of tranquilizers and/or antidepressants (continued to use tranquilizers and/or antidepressants, started taking tranquilizers and/or antidepressants, increased the dosage they took of tranquilizers and/or antidepressants). It should be noted that all variables and their response categories were dichotomized (0 and 1) to be entered into the binary logistic regression model.

Two logistic regression analyses were performed; the first considering as dependent variable people who considered their mental health condition as poor. The second regression analysis considered as dependent variable people who reported changes in relation to their mental health during the pandemic period (feeling more agitated, anxious, sad, irritated, discouraged, crying more easily, lonely, always thinking about COVID-19, work overload and/or sleeping difficulties, when compared to the pre-pandemic period).

Thus, based on the knowledge of the authors and the literature, variables that may be related in any way to mental health and/or emotional changes, such as the use of tranquilizers or antidepressants, were included in the study, in order to verify whether there was any change in use or association with poor mental health status.

Exploratory analysis was conducted for collinearity among the independent variables using the Variance Inflation Factor (VIF), and those with values greater than 10 [22] were removed from statistical modeling. The modeling was performed using the backward stepwise selection method, in which one starts with a complete model (with all variables) and then removes the variables one by one and verifies the model’s behavior. The best model considered was the one with the lowest Akaike Information Criterion (AIC) value [23]. It is also worth mentioning that for the final model, the Odds Ratio (OR) with their respective 95% Confidence Intervals (95%CI) were calculated.

After exhausting all possibilities of analysis and choosing the final model (based on the criterion of lowest AIC value), the Hosmer-Lemeshow, likelihood ratio, CoxSnell, Nagelkerke, and McFadden tests were performed to validate the model. In addition, the predictive ability and accuracy of the models were checked based on the area under the Receiver Operating Characteristic curve (ROC curve) and their respective 95% CI values [24]. The validation analyses and tests were performed using RStudio software.

### Ethical aspects

The entire conduct of the research occurred in accordance with Resolution No. 466 of December 12, 2012 of the National Health Council, meeting the relevant ethical and scientific criteria. Participation began only after reading and accepting the Informed Consent Form (ICF), which was made available when the participant accessed the questionnaire. The parent study was approved by the Research Ethics Committee of ENSP-FIOCRUZ (CAAE: 32210320.1.0000.5240) and of EERP-USP (CAAE: 32210320.1.3001.5393), the latter due to the research partnership.

## Results

A total of 2,698 people answered the questionnaire, 1380 in 2020, 834 in 2021 and 484 in 2022. The majority of respondents being female (n = 1,667, 61.7%), age range between 18 and 39 years (n = 1,187, 44.0%), with 18 years as the minimum age and 89 years as the maximum, mean age 44 years, median 42 years, and standard deviation 15 years. The predominant race/color was white (n = 1,463; 54.2%), married, widowed and separated or single (n = 1,632; 60.5%), and with complete college education (n = 1,989; 73.7%). The majority declared to be formal workers (n = 1,109; 41.1%), with income between 1 and 5 minimum wages (n = 860; 31.9%) and declared not to receive any type of government assistance (n = 2,438; 90.4%), In addition, (n = 714; 40.2%) declared having been infected with COVID-19 and (n = 883; 32.7%) received at least one dose of vaccine, as presented in Table 1.

**Table 1:** Sociodemographic profile of the participants under study, Brazil, 2022 (N = 2,698).

Variables	N (%)
Sex	
Male	1004 (37.2%)
Female	1667 (61.7%)
Other	14 (0.5%)
Ignored	13 (0.5%)
Age group	
18 to 39 years old	1187 (44.0%)
40 to 59 years old	1013 (37.5%)
60 years or more	498 (18.5%)
Race/color	
White	1463 (54.2%)
Brown/Black	1113 (41.3%)
Other	75 (2.8%)
Ignored	47 (1.7%)
Marital Status	
Married or Stable Union	1054 (39.1%)
Widowed, separated or single	1632 (60.5%)
Ignored	12 (0.4%)
Education	
No education	12 (0.4%)
Elementary school complete	357 (13.2%)
High school complete	323 (12.0%)
College or post-graduation	1989 (73.7%)
Ignored	17 (0.6%)
Occupation	
Formal work	1109 (41.1%)
Informal work	548 (20.3%)
Unemployed	124 (4.6%)
Student	312 (11.6%)
Ignored	605 (22.4%)
Monthly income	
No income	298 (11.0%)
Less than 1 minimum wage	236 (8.7%)
From 1 to 5 minimum wages	860 (31.9%)
From 5 to 10 minimum wages	539 (20.0%)
Above 10 minimum wages	566 (21.0%)
Ignored	199 (7.4%)
Receives government assistance	
Yes	260 (9.6%)
No	2438 (90.4%)
Covid-19	
Yes	714 (40.2%)
No	1060 (59.8%)
Ignored	923 (34.2%)
Vaccination	
Yes	883 (32.7%)
No	46 (1.7%)
Ignored	1768 (65.6%)

**Fig 1:**
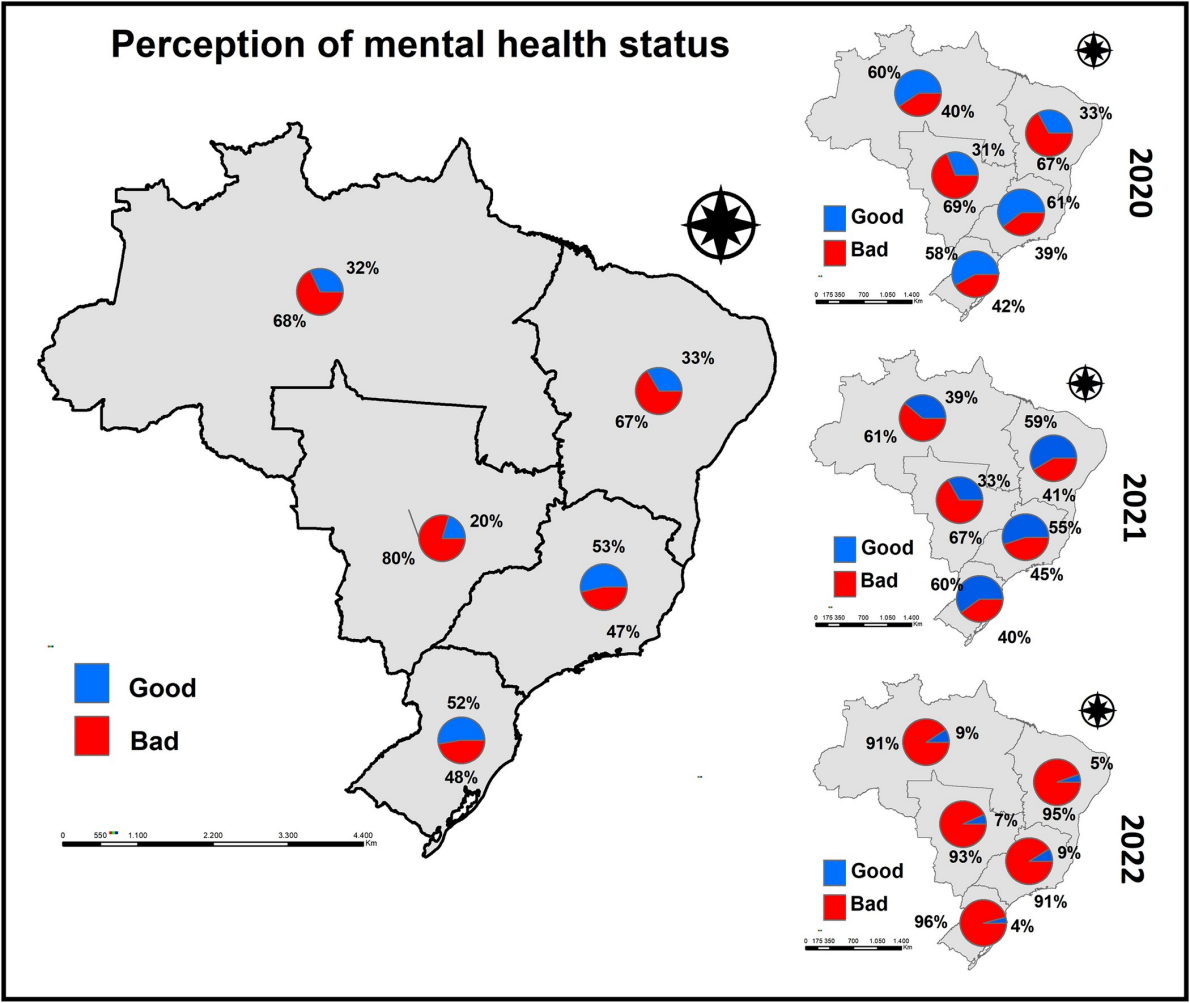
Percentage of self-perception of mental health of individuals considering the place of residence according to the five macro-regions of the country, Brazil, 2022. The Shapefile with the division of the macro-regions of Brazil was obtained free of charge through the IBGE website: https://www.ibge.gov.br/geociencias/organizacao-do-territorio/malhas-territoriais/15774-malhas.html.

The negative self-perception of mental health showed a heterogeneous spatial distribution in the five macro regions of Brazil (North, Northeast, Center-West, Southeast, and South). Fig 1 shows the individual perception of mental health at the moment they answered the questionnaire, also separated by years, in order to verify differences according to the course of the pandemic. Thus, it can be observed that the highest percentage of people who classified their mental health as "good" lived in the Southeast (53%) and South (52%) regions of Brazil, while those who declared it as "poor" lived in the North (68%), Northeast (67%), and Center-West (80%) regions.

When separated by year, it is observed that in 2020, the pre-vaccine period, in the North, Southeast, and South regions of Brazil, people mostly classified their mental health as "good" (60%, 61%, and 58%, respectively), while the Central-West and Northeast regions presented the highest percentages of people who classified their mental health as "poor" (69% and 67%, respectively). In 2021, the period in which vaccination began, the Northeast (59%), Southeast (55%), and South (60%) regions of Brazil remained with the majority of people who classified their mental health as good, while in the North (61%) and Central -West (67%) regions of the country, people classified their mental health as "poor". Finally, a completely different scenario is observed when we separate the year 2022, in which the absolute majority of all regions of Brazil classified their mental health as "poor".

**Fig 2:**
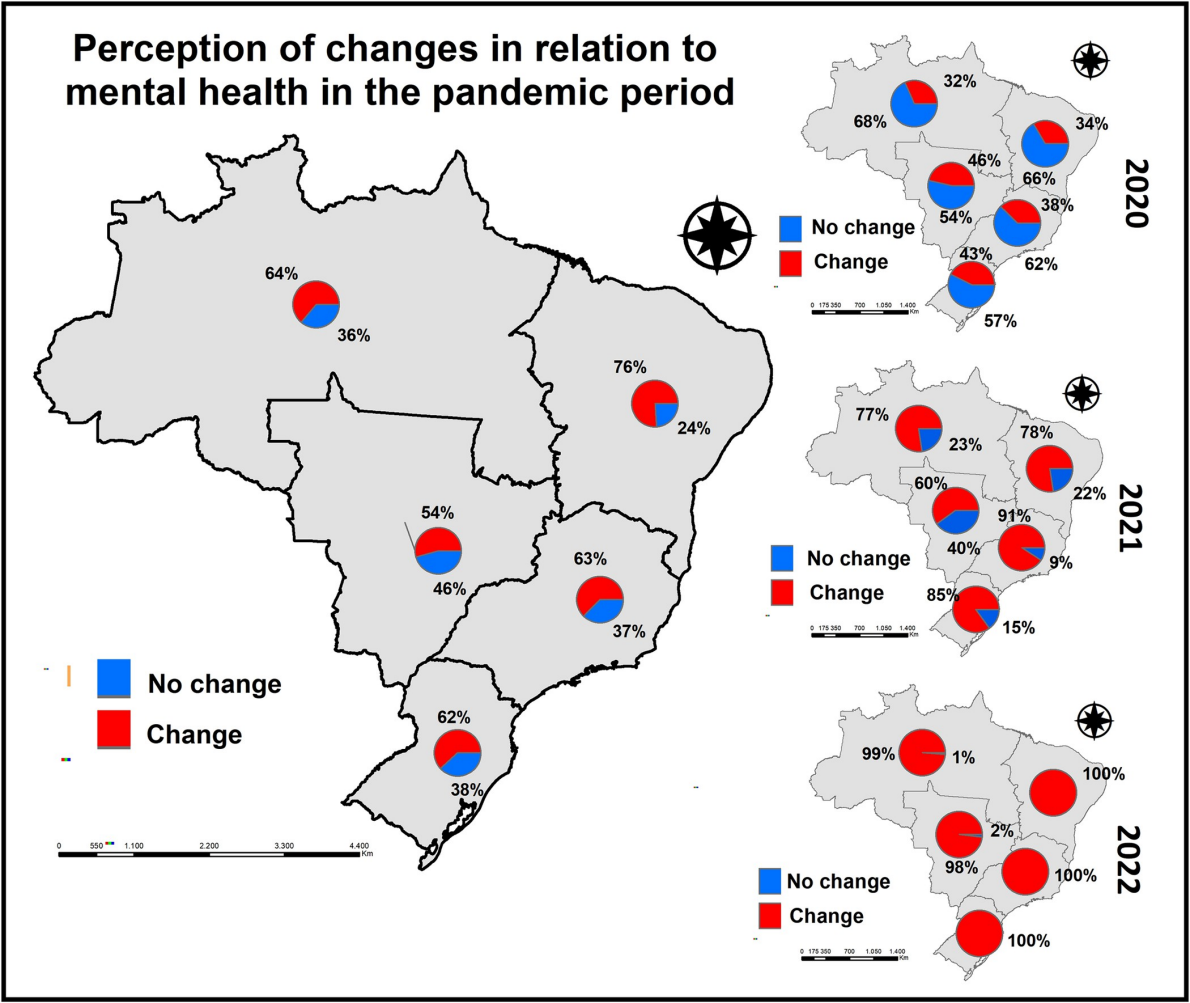
Percentage of self-perception of changes in relation to mental health in the pandemic period considering the place of residence according to the five macro-regions of the country, Brazil, 2022. The Shapefile with the division of the macro-regions of Brazil was obtained free of charge through the IBGE website: https://www.ibge.gov.br/geociencias/organizacao-do-territorio/malhas-territoriais/15774-malhas.html.

Fig 2 refers to the perception of change regarding mental health during the pandemic period compared to the pre-pandemic period. It is worth noting that regarding mental disorders/emotional problems, we observed a higher prevalence of feeling more agitated, anxious, sad, irritable, demotivated, crying more easily, feeling more lonely, always thinking about COVID-19, greater work overload and/or difficulty sleeping compared to before the start of COVID-19. Thus, it is possible to observe that in all macro regions of the country, the highest percentage of respondents reported changes in their self-reported mental health, with the highest percentages observed in the Northeast and North regions of Brazil (76% and 64%, respectively).

When separated by year, we observed that the percentages related to changes in mental health gradually increased during the COVID-19 pandemic. In 2020, we observed that in the North, Northeast, and Southeast regions of Brazil, most people did not report changes (68%, 66%, and 62%, respectively), while the Central-West and South regions presented the highest percentages of change in mental health (46% and 43%, respectively). In 2021, the majority of respondents from all regions reported changes in mental health, with the highest proportions observed in the Southeast (91%), South (85%), North (77%), and Northeast (76%) regions. Finally, in 2022, the absolute majority of all regions of Brazil reported changes in mental health, reaching 100% of respondents from the Northeast, Southeast, and South regions of Brazil.

To validate the model presented in Table 2, it was verified that the accuracy capacity of the model through the area under the ROC curve showed a value of 0.83, besides the Hosmer-Lemeshow test (p = 0.16), likelihood ratio (p = <0.01), CoxSnell (0.30), Nagelkerke (0.41) and McFadden (0.26).

**Table 2:** Characteristics associated with people who considered their mental health status as bad.

Variables	Coefficient	Valor p	Odds Ratio [IC95%]
Age: 18 to 39 years old	-0.99	<0.01*	0.37 [0.24–0.56]
Age: 40 to 59 years old	-0.33	0.10	0.71 [0.47–1.07]
Race/color: Black	-0.07	0.62	0.92 [0.67–1.27]
Occupation: Formal worker	0.16	0.27	1.18 [0.87–1.58]
Education: College degree	0.68	<0.01*	1.97 [1.21–3.22]
Felt calm or cheerful as a result of isolation measures	-10.96	<0.01*	0.33 [0.14–0.68]
Compared to before the COVID-19 pandemic, felt less agitated, anxious or tense	-0.62	<0.01*	0.53 [0.39–0.72]
Compared to the COVID-19 pandemic, felt less irritable	-0.44	<0.01*	0.64 [0.46–0.88]
Compared to before the COVID-19 pandemic, felt less sad, less discouraged or cried less easily	-10.46	<0.01*	0.35 [0.25–0.47]
Compared to before the COVID-19 pandemic, felt less overwhelmed	-0.23	0.13	0.78 [0.27–1.07]
Compared to before the COVID-19 pandemic, can now accomplish all tasks	-0.26	0.09	0.76 [0.56–1.04]
Compared to the COVID-19 pandemic, no problems sleeping	-0.75	<0.01*	0.46 [0.34–0.64]
Compared to the COVID-19 pandemic, no change in their feelings	0.59	0.12	1.80 [0.88–4.01]
Compared to before the COVID-19 pandemic, unchanged its consumption of over-processed foods	-0.24	0.12	0.77 [0.56–1.07]
People who reported using tranquilizers or antidepressants to cope with the current situation	0.71	<0.01*	2.04 [1.46–2.85]
People who have not started or increased their use of tranquilizers or antidepressants as a result of the pandemic	-18.88	<0.01*	0.15 [0.04–0.44]
Not being a worker from a risk group for contamination by COVID-19	-0.39	0.01*	0.67 [0.49–0.91]

*p<0.05.

In the multicollinearity analysis, variables referring to males and females were excluded, as they had a VIF value >10.

In Table 2, it was possible to identify the factors associated with self-reported mental health and emotional change. It was found that people aged 18 to 39 years were less likely [OR: 0.37; 95%CI: 0.24–0.56] to consider their mental health status poorly compared to other age groups, as these were people who reported being less likely to feel calm and cheerful as a result of the isolation measures (OR: 0.33; 95%CI: 0.14–0.68).

Compared to the pre-pandemic period of COVID-19, people who reported feeling less agitated, anxious and tense were less likely (OR: 0.53; 95%CI: 0.39–0.72) of considering their mental health status poorly compared to the post-pandemic period. The same is true for people who reported feeling less irritable (OR: 0.64; 95%CI: 0.46–0.88), less sad, despondent or crying less easily (OR: 0.35; 95%CI: 0.25–0.47) and having no problems sleeping (OR: 0.46; 95%CI: 0.34–0.64).

Also as protective factors, it was identified that people who did not start or increase the use of depressants or antidepressants because of the pandemic have fewer chances (OR: 0.15; 95%CI: 0.04–0.44) to consider their mental health status bad, as well as not being workers from risk groups for contamination by COVID-19 (OR: 0.67; 95%CI: 0.49–0.91).

Regarding risk factors, it was identified that people with complete higher education (OR: 1.97; 95%CI: 1.21–3.22) and people who reported using tranquilizers or antidepressants to cope with the current situation (OR: 2.04; 95%CI: 1.46–2.85) were more likely to consider their mental health status poorly.

In the second regression analysis carried out, we considered as a dependent variable those people who reported changes in relation to their mental health during the pandemic period, which the research participants could consider as changes, according to the questionnaire response options that were chosen. feel more agitated, anxious, sad, irritated, discouraged, cry more easily, lonely, always thinking about COVID-19, work overload and/or difficulty sleeping, seeking to identify factors related to a negative emotional change.

To validate the model presented in Table 3, it was verified that the accuracy capacity of the model through the area under the ROC curve presented a value of 0.78, besides the Hosmer-Lemeshow test (p = 0.52), likelihood ratio (p = <0.01), CoxSnell (0.12), Nagelkerke (0.24) and McFadden (0.17).

**Table 3:** Characteristics associated with people who showed worsening in their mental health in the pandemic period compared to the pre-pandemic period.

Variables	Coefficient	Valor p	Odds Ratio [IC95%]
Education: elementary school completed	-14.14	<0.01*	0.24 [0.10–0.58]
Education: high school completed	-11.83	<0.01*	0.30 [0.15–0.60]
Received some kind of government assistance	0.68	0.06	1.99 [-1.00–4.21]
Increased consumption of ultra-processed food	0.91	<0.01*	2.49 [1.43–4.61]
Increased consumption of alcoholic beverages	0.69	0.07	2.00 [0.98–4.66]
People who reported using tranquilizers or antidepressants to cope with the current situation	-0.50	0.11	0.60 [0.31–1.09]
People who have not started or increased their use of tranquilizers or antidepressants as a result of the pandemic	12.63	0.10	3.53 [0.91–23.39]
People who have not started or not increased the use of calming or antidepressant medications as a result of the pandemic	-19.85	<0.01*	0.13 [0.03–0.53]
People who considered their mental health status to be poor	-22.92	<0.01*	0.10 [0.04–0.18]
Not being a worker from a group at risk for contamination by COVID-19	0.31	0.14	1.36 [0.90–2.09]

*p<0.05.

In the multicollinearity analysis, the variables that presented VIF>10 were excluded, as follows: female and male sex; people of white and black/brown race/color; people who declared that they have been feeling more agitated, anxious or sad due to the isolation measures; people who declared, compared to the period before COVID-19, to be more agitated, anxious or tense; more irritated; more sad, discouraged or cry more easily; feel more lonely; are more burdened; they can’t do everything they have to do; had more difficulty sleeping and are always thinking about COVID-19.

In Table 3, it was possible to identify four protective and one risk variable. We identified that individuals who had elementary school (OR: 0.24; 95%CI: 0.10–0.58) or complete high school (OR: 0.30; 95%CI: 0.10–0. 60) were less likely to report changes in their mental health status in the pandemic period compared to the pre-pandemic period. Also, people who did not start or increase their use of depressants and/or antidepressants because of the pandemic (OR: 0.13; 95%CI: 0.03–0.53) and those who considered their mental health status poor (OR: 0.10; 95%CI: 0.04–0.18) were identified. In turn, people who reported increased consumption of ultra-processed foods (OR: 2.49; 95% CI: 1.43–4.61) were more likely to report changes regarding their mental health in the pandemic period compared to the pre-pandemic period.

## Discussion

The COVID-19 pandemic has triggered the need for measures by federal, state and municipal governments around the world to contain the virus and prevent transmission. The closure of institutions and businesses was essential to reduce the manipulation of the virus, however, leading to emotional disorder in well-being. In this sense, social isolation measures had its consequences reflected on mental health in the general population [24].

The objective of the present study was to identify factors associated with self-perceived mental health changes while facing the COVID-19 pandemic in Brazil. People with impaired mental health had a lower prevalence of situations such as being less agitated, anxious, sad, angry, discouraged and crying less easily. Some subjects were also less likely to feel less overworked and/or have trouble sleeping.

The results found in this study are similar to studies developed in India, United Kingdom, Germany and China that the pandemic was indicated as a negative factor for the mental health of the general population. Negatively affecting all aspects of individuals’ lives and leaving them more vulnerable to the development of mental disorder problems, it can be seen that the pandemic has caused mental and physical damage, as well as leading to psychological conditions of medium and severe intensity, such as post-traumatic stress disorders, depression, anxiety, panic disorders and behavioral disorders [25–28].

We also verified that among the resources to mitigate emotional problems and relief anxiety was to resort to self-medication and alcohol use. We identified that people with complete college education (OR: 1.97; 95%CI: 1.21–3.22) and people who reported using tranquilizers or antidepressants (OR: 2.04; 95%CI: 1.46–2.85) self-reported better health status. Income was a marker for people’s access to these resources, which somewhat highlights disparities among the groups studied.

The findings were quite elucidative, in a way that the collection of information via web survey allowed a heterogeneous participation of the Brazilian population, involving people from all the five macro-regions of Brazil, of different social groups, ages, and with different levels of education.

Thus, the incorporation of coping strategies can directly contribute and impact the psychological responses and mental health of the population, that is, protective and/or harmful effects on mental health. Strategies such as social support, positive thoughts, acceptance, purpose in life, good humor to face daily adversities and planning are factors that contribute to the reduction of stress, anxiety and perceived depression. In this sense, it is necessary for health professionals to act in a holistic way centered on the individual and to meet the biopsychosocial needs of the population [29].

However, the sociodemographic characteristics of this study are not representative of the general Brazilian population; this can be explained by the method used for data collection itself, which is based mainly on greater access to the Internet and familiarity with digital platforms by the people who answered the survey. In the literature it is already reported that online survey presents a greater sample predominance of individuals who have easier access to the internet [30,31], such as young people with better social and economic conditions- who are those who mostly use the internet and social networks [32].

The findings also evidenced a higher number of female respondents than male, this relationship can be verified in other studies that used the online survey method [24,33,34]. A survey study conducted by researchers from Fundação Getúlio Vargas with social assistance professionals in Brazil, showed that 85.7% of the people who answered the questionnaire were female and only 12% were male [35], besides the fact that women are more concerned about health issues [36].

In the Midwest and South regions of Brazil, we observed the highest percentage of people who classified their mental health as "good", but they were the ones who perceived more changes in relation to their mental health in the pandemic period, compared to the pre-pandemic period. However, it is emphasized that people with their mental health status considered good also deserve attention, since uncertainty about the disease, apprehension about death, or fear of infection from family and friends can be the trigger to changes in their mental health status, and even lead to the development of mental disorders [37–39].

In the pre-pandemic period, populations were oriented on the forms of transmission or development of the COVID-19 disease, the first stage of the disease was associated with information on mortality in elderly people and individuals with Chronic Noncommunicable Diseases. The general characteristics were social distancing information, and risk of infection by direct contact with sick individuals. Planning was essential for assessing the risk of the disease, based on clinical consultations [40].

A very important aspect that contributed to negative emotions, as well as the development of anxiety associated with COVID-19, was the news of deaths and the severity of the disease, often without scientific substantiation, directly affecting mental health and panic syndromes in the population. general. These distorted predictions triggered negative emotions throughout the pandemic–such as sadness, anguish and fear. It is worth mentioning that mental health at the beginning of the pandemic was not something visible by health managers, since the concern was to contain transmission and the level of contamination of the population [41].

The assumption of keeping people out of contact with other people at the beginning of the pandemic was fundamental to mitigate the contagion of the disease and, consequently, to reduce the demand for health services. In the last stage of the pandemic, there was a decline in new cases and a decrease in transmission of the virus, social distancing measures were eased, and the outbreak was under control. People resumed their usual activities, gradual return of trade, and institutions. However, the pandemic brought consequences on mental health that were not considered urgent due to the increase in hospitalized people, large-scale transmission, increase in deaths from the disease, revealing little, or almost no, attention to mental health issues [42].

Studies have shown an increase in mental disorders at the beginning, during and at the end of the pandemic. The study found that, post-pandemic, about 52,000 people residing in China, had moderate and severe psychological sequelae. People over 40 years of age, with a higher educational level, were the most vulnerable to depression, stress, anxiety, compulsive behavior and impairments in social behavior. Linked to this phenomenon, there is concern about the efforts of public health authorities on the psychological effects of the disease. As we worked throughout the study, the mental health repercussions that the pandemic scenario caused on people with specific vulnerability cannot be overemphasized. Encompassing actions aimed at alleviating mental suffering throughout the COVID-19 crisis [43].

Grolli [44], reiterate that age also arises in the involvement of emotional and psychological changes, changes associated with age become factors causing depression, anxiety disorders, stress and distress. In this context, regarding the problem of the decaying connection of the COVID-19, populations older than 40 years had higher negative feelings from the pandemic. This population is also suffering from inherent uncertainties and social isolation associated with the pandemic, corroborating with what was observed in the present study when people aged between 18 and 39 years had lower chances of considering their mental health condition as bad.

With regard to age, the results indicate a lower risk of mental disorders in younger people, fear of contracting the virus, fear of loss and death and sequelae of medical complications, factors that may indicate problems in the mental health of the population with age greater than 40 years. In addition, the impact provided by the pandemic on the mental health of this population, since public policies only classify chronic diseases such as Diabetes Mellitus and Systemic Hypertension as a problem. In addition to the association of age with immunosenescence, psychiatric disorders can aggravate the immune system [45].

In a literature review [46] that sought to synthesize the mental health outcomes arising from the COVID-19 pandemic, a number of psychological responses that people began to exhibit after the emergence of COVID-19 and at the beginning of the pandemic were described. The main ones being anxiety, depression and stress, insomnia, concern about one’s own health and family, difficulty socializing and going out in crowded places, dissatisfaction with life, phobias, compulsive behavior, physical symptoms, and impaired social functioning.

The fact that a good number of individuals feel calmer or happier as a result of the physical isolation measures is intriguing, since psychological manifestations tend to be prevalent in people who are in physical isolation or quarantined, these are times when psychological distress tends to be greatest [47]. During physical isolation or quarantine, people may commonly have even more intense responses, such as fear, boredom, loneliness, anxiety, insomnia, and anger as seen in confirmed or suspected COVID-19 patients [48]. These psychosomatic manifestations deserve attention, both by family members and also by those close to them, since they may evolve into more serious disorders, such as panic attacks and post-traumatic stress, severe depressive crises or psychotic outbreaks, which may even lead to suicide [30,38,49].

Public policy relationships emerge to explain the degrees of psychological disorders and their problems in subpopulations with and without infection. It is assumed that populations may show psychopathological symptoms due to several reasons: mismanagement in the representation of public policies, uncertainties in journalistic presentation in media coverage, physical discomfort and fear of transmission of the virus. The findings suggest that the psychological impact on populations during the COVID-19 pandemic is significant, leading to lower quality of life and higher prevalence of stress attributed to all stages of the disease [50].

When discussing the relationship of individuals with their residential environment in a physically distant situation in Brazil during the pandemic, one study signaled that physical distancing with prolonged stay inside the home was a source of stress for most participants in the country. The authors believe that such stress occurs mainly due to mobility restrictions, intensification of family interaction caused by the situation of being confined for several months [51].

Studies show that, in a situation of social isolation and/or local quarantine, some behaviors of discomfort are common, such as feelings of powerlessness, sadness, loss of sleep, excessive consumption of alcohol and ultra-processed foods. Emotional and behavioral changes were associated with increased time of connivance at home, overload due to multiple household tasks and less availability of access to public and health services. Still according to the study, the search for mental health care must occur effectively to the needs of the population. If, on the one hand, the severity of psychiatric disorders is reported, there is concern about care in the face of poor management of public policies in the care of this population [52].

A longitudinal study that assessed the impact of lockdown on mental health during the first year of the COVID-19 pandemic in a general sample of the French population identified that such a health crisis strongly affected the occurrence and persistence of depression, anxiety, and post-traumatic stress disorder over time [2]. The prevalence of depression symptoms among adults in the United States increased dramatically during the early months of the COVID-19 pandemic. A study that sought to understand the impact of the pandemic on people with a history of depression showed that this relationship increased the chances of negative effects of the pandemic on various aspects of life [3].

The pandemic scenario generated several inequalities, including the scope of work, especially when observed in services that are considered essential to society, such as health professionals, gravediggers, police officers, workers in pharmaceutical and food establishments, among others. These group of essential workers presented greater exposure to various difficulties during the crisis. However, certain groups of workers, such as commercial and industrial workers, housekeepers, truck drivers, and bricklayers have recorded an elevated number of cases of COVID-19 infection. This exposure is due to various reasons, such as in Italy, where supermarket cashiers reported that gloves, disinfectant gel, and only one pair of masks were available, and that reused ones would be necessary [53]. Also in São Paulo, the Labor Public Ministry (MPT) reported that there were approximately 500 complaints between March 1 and 24, 2020, from employees against companies reporting the risk of contamination (Rede Brasil Atual, 2020) [54].

In this sense, the findings of this study reaffirm that not belonging to the class of workers in the risk group for contamination by COVID-19 reduces the negative repercussions on mental health status. This is a fact, considering that contamination by COVID-19 can develop immediate effects of anxiety and stress among workers, as observed in a study, which found that 39% of health professionals presented some psychological distress, especially those who worked with excessive workload in Wuhan [55].

This study also identified having completed higher education and using tranquilizers or antidepressants to cope with the pandemic distress as risk factors for poor mental health. Fear, distress, and sadness were manifestations that were present during the pandemic and led to various psychological repercussions related to physical detachment, stress, and unemployment. In the literature, association of impacts of the pandemic with the use of tranquilizer and antidepressant medications, whether rationally or not is presented, considering the stressors that aggravate mental disorders [56]. It is worth mentioning that the COVID-19 pandemic had a high potential for contagion and considerably induced the fear of virus transmission especially in individuals who have higher levels of education, because they are the ones who have more access to information [27].

It is also found that the protective factors and risks for self-reported mental health identified in our study resembles in part the evidence stating that young people- those with pre-existing mental health conditions, and the financially disadvantaged, experienced greater declines in mental health [57]. This makes one reflect on the diversity of the affected settings and populations and the importance of biopsychosocial aspects of the social determinants of mental health at the time of the pandemic.

Thus, considering several intrinsic aspects (self-determination/aspirations) and extrinsic aspects (biopsychosocial and environmental aspects) one cannot minimize the psychological repercussions that the general scenario of the pandemic causes on individuals, specific groups, and society as a whole. According to representatives of psychology in Brazil, the very impact of this health emergency on people’s mental health can be a limiting factor for the country to overcome the COVID-19 crisis [42].

Another factor that was observed before the COVID-19 pandemic is the risk of people with mental disorders developing unhealthy behaviors [58] such as bad eating habits. One study found that individuals with depression were 49% more likely to consume ultra-processed foods compared to those without depression [59]. A study from the NutriNet Brazil cohort verified a tendency to increase the consumption of over processed foods in the northern macro-region of the country and among the lower education category [60]. Our study corroborates with the findings of this study, as people who reported increased consumption of ultra-processed foods were those more likely to report changes in relation to their mental health during the pandemic compared to the pre-pandemic period. These results point to a reduction in the healthy eating habits and quality of life of this population during the pandemic, directly affecting mental health.

Biomedical and epidemiological strategies should be combined with contributions of psychological knowledge, as this would avoid greater repercussions on the health, quality of life and well-being of the population, while achieving greater effectiveness in public health strategies [9].

Adaptive coping initiatives seem to have been protective and helped regulate symptoms of mental problems, constituting a possible approach to mitigate negative effects, since they promote well-being, relieve stress, and reduce perceptions of helplessness.

Besides the multiple implications that involve the process of coping and containment of a pandemic outbreak, it is important to guarantee the population appropriate mental health care, encompassing actions aimed at alleviating mental suffering throughout the crisis [49].

### Limitations

As limitations of the study, we highlight the study design employed. Since this was an online survey, several social segments were not included (such as people in situations of social vulnerability). Barrier of access to the Internet may have caused the involuntary exclusion of these groups and influenced the estimates of the proportion of responses, justifying the difference between the profiles of the study sample compared to the Brazilian population. The analysis of self-reported conditions rather than formal mental health diagnoses is a limitation to be considered in this work. Thus, it is important that further studies be conducted to understand the real impact that the COVID-19 pandemic may have caused on the mental health of the Brazilian population.

It also has the following limitations regarding snowball sampling: like all non-probabilistic sampling, it is not “representative” and generalizations cannot be made about a population it may also be that the study participants are not very diversified, since the participant tends to indicate the research to other people who are within the social cycle of the indicating member and/or researchers involved [61].

In addition, we highlight that the impacts on the mental health of the population caused by the COVID-19 pandemic may involve other aspects not considered in the analyses, such as fear of contagion, deaths of close people and COVID-19 itself, in addition to concerns about policies of protection to the population and also related to vaccination.

The analysis of self-reported conditions rather than formal mental health diagnoses is a limitation to be considered in this work. Thus, it is important that further studies be conducted to understand the real impact that the COVID-19 pandemic may have caused on the mental health of the Brazilian population.

## Conclusion

The study showed knowledge about the consequences of the pandemic on the mental health of the Brazilian population, and brought the sociodemographic and spatial profile of the study participants. It identified self-perception and factors associated with mental health during the COVID-19 health crisis.

People have been found to have suffered significant mental distress, including anxiety, depression and traumatic symptoms at the beginning, during and at the end of the COVID-19 pandemic. In addition, the results appreciated that the pandemic had serious consequences on the mental health of the Brazilian population. In addition, some variables were associated with greater mental impact, such as being female and being older. High rates of negative mental health were also observed, including symptoms of stress, anxiety and overeating. Therefore, these results reinforce the idea that public mental health interventions should be formally built into public health emergency preparedness and response plans.

With regard to people experiencing COVID-19, interventions should be based on a comprehensive assessment of the risk factors that lead to psychological problems, including poor mental health before a crisis, bereavement, personal or family injuries, circumstances that life-threatening, panic, family separation and low-income.

These measures may help to decrease or prevent future psychiatric morbidities. In addition, public policies associated with primary health care should be invested in, so that early signs and symptoms associated with mental disorders are observed so that early interventions to be developed.

## Supporting information

S1 File STROBE statement—checklist of items that should be included in reports of observational studies. (PDF)

S1 Data Minimal anonymized data set. (XLSX)

S1 Text (TXT)

S2 Text (TXT)
