# Supplementary material for: Factors associated with status and self-perceived mental health changes in the face of the COVID-19 pandemic in Brazil
Source: PLOS Glob Public Health. 2023 Aug 18;3(8):e0001636. doi: 10.1371/journal.pgph.0001636 (PMC10437945; doi:10.1371/journal.pgph.0001636)
Supplement: S1 File — (PDF) [file pgph.0001636.s001.pdf]

STROBE Statement—Checklist of items that should be included in reports of ***cross-sectional studies***

	Item No	Recommendation	Page No
**Title and abstract**	1	(*a*) Indicate the study's design with a commonly used term in the title or the abstract	Pág.1 FACTORS ASSOCIATED WITH STATUS AND SELF-PERCEIVED MENTAL HEALTH CHANGES IN THE FACE OF THE COVID-19 PANDEMIC IN BRAZIL.
		(*b*) Provide in the abstract an informative and balanced summary of what was done and what was found	Pág.2 **Background:** The Public Health Emergency of International Importance triggered by SARS-CoV-2 has greatly triggered emotional problems and mental health. The objective of this study is to analyze the perceived change in mental health/psychic and its determinants in Brazil. **Methods:** Cross-sectional study, conducted among 2,698 Brazilians and foreign migrants living in Brazil, aged 18 years or older. The analysis was based on descriptive statistics and logistic regression. **Results:** The sample consisted mostly of whites, constituting 61.7% of women. We found that people with complete college education and people who reported using tranquilizers or antidepressants to cope with their current situation were 1.97 (95%CI: 1.21 - 3.22) and these group were 2.04 (95%CI: 1.46 - 2.85) times more likely to consider their mental health status as poor. People who reported to have increased consumption of ultra-processed foods compared to their consumption of such foods in the pre-pandemic period were 2.49 more likely [95%CI: 1.43 - 4.61] to report changes regarding their mental health in the post-pandemic period. Participants also reported more difficulty sleeping (95% CI: 0.34 - 0.64). **Conclusion:** The results showed the presence of mental disorders among the population due to the impact of the COVID-19 pandemic as well as greater reporting of emotional problems. High self-medication consumption was observed in socially- advantaged groups.
**Introduction**			
Background/rationale	2	Explain the scientific background and rationale for the investigation being reported	Pág. 2 – paragraph 1. The COVID-19 pandemic had immeasurable impacts on the lives and health of people around the world. Since 2020, the Pan American Health Organization (PAHO) has warned about the increase in mental suffering caused by the emergence of the new coronavirus. This is due to the strategies used for its

			containment, such as physical isolation, as the various containment measures put in place have affected the mental wellbeing of the population, causing an increase in psychological suffering and psychic symptoms. Pág. 3 – paragraph 3. The onset of mental disorders depends on complex mechanisms such as neuro functional factors, exposure to environmental stressors, and biological susceptibility (epigenetics). It is worth noting that environmental factors can alter gene expression, so, exposure to extremely unfavorable environmental conditions associated with increased social vulnerability, further exacerbated by the COVID-19 pandemic, can trigger mental disorders even in individuals without genetic predisposition, and this may explain the epidemic potential for post-pandemic mental health changes. Pág. 4 – paragraph 1. Although there is a dense body of published work on this topic in the literature, more mental health research needs to better specify and explain the heterogeneous factors identified, as this heterogeneity contributes to significant differences in the prevalence of reported mental health symptoms. There is a need to advance the knowledge of mental health and factors associated with it in the face of the health crisis experienced in Brazil. In this context, the aim of this study was to identify factors associated with self-perceived mental health changes while facing the COVID-19 pandemic in Brazil.
Objectives	3	State specific objectives, including any prespecified hypotheses	Pág. 4 – paragraph 1. There is a need to advance the knowledge of mental health and factors associated with it in the face of the health crisis experienced in Brazil. In this context, the aim of this study was to identify factors associated with self-perceived mental health changes while facing the COVID-19 pandemic in Brazil.
**Methods**			
Study design	4	Present key elements of study design early in the paper	Pág. 4 – paragraph 2. This is a cross-sectional, descriptive and analytical study [11], with data collection via web survey. The study covered 26 units of the federation and the Brazilian Federal District, in the period between August 2020 to November 2022.
Setting	5	Describe the setting, locations, and relevant dates, including periods of recruitment, exposure, follow-up, and data collection	Pág. 4 – paragraph 2, 3 and 4. This is a cross-sectional, descriptive and analytical study , with data collection via web survey. The study covered 26 units of the federation and the Brazilian Federal District, in the period between August 2020 to November 2022.

			The study population was made up of people who declared that they were Brazilians or foreign migrants who understood the language spoken in Brazil (Brazilian Portuguese) and had lived in Brazil for at least six months, 18 years of age or older, and who were willing to participate in the survey- having internet access available.<br>As for the sampling design, the study was based on a convenience sampling, using the snowball technique, which is characterized by the non-adoption of error margin calculations, since it is a non-probabilistic method. Therefore, we used the calculation for finite populations , arriving at a minimum sample size of 1,428 participants.
Participants	6	(*a*) Give the eligibility criteria, and the sources and methods of selection of participants	Pág. 4 – paragraph 3 and 4. The study population was made up of people who declared that they were Brazilians or foreign migrants who understood the language spoken in Brazil (Brazilian Portuguese) and had lived in Brazil for at least six months, 18 years of age or older, and who were willing to participate in the survey- having internet access available.<br>As for the sampling design, the study was based on a convenience sampling, using the snowball technique, which is characterized by the non-adoption of error margin calculations, since it is a non-probabilistic method. Therefore, we used the calculation for finite populations [12], arriving at a minimum sample size of 1,428 participants.
Variables	7	Clearly define all outcomes, exposures, predictors, potential confounders, and effect modifiers. Give diagnostic criteria, if applicable	Pág. 4 – paragraph 3,4,5 and 6. To identify factors associated with the outcomes of interest, binary logistic regression was used based on the variables present on the "Social Thermometer" instrument (Questionnaire), which were dichotomized.<br>Two logistic regression analyses were performed; the first considering as dependent variable people who considered their mental health condition as poor. The second regression analysis considering as dependent variable people who reported changes in relation to their mental health during the pandemic period (feeling more agitated, anxious,

			sad, irritated, discouraged, crying more easily, lonely, always thinking about COVID-19, work overload and/or sleeping difficulties, when compared to the pre-pandemic period).<br>Exploratory analysis was conducted for collinearity among the independent variables using the Variance Inflation Factor (VIF), and those with values greater than 10 [19] were removed from statistical modeling. The modeling was performed using the backward stepwise selection method, in which one starts with a complete model (with all variables) and then removes the variables one by one and verifies the model's behavior. The best model considered was the one with the lowest Akaike Information Criterion (AIC) value [20]. It is also worth mentioning that for the final model, the Odds Ratio (OR) with their respective 95% Confidence Intervals (95%CI) were calculated.<br>After exhausting all possibilities of analysis and choosing the final model (based on the criterion of lowest AIC value), the Hosmer-Lemeshow, likelihood ratio, CoxSnell, Nagelkerke, and McFadden tests were performed to validate the model. In addition, the predictive ability and accuracy of the models were checked based on the area under the Receiver Operating Characteristic curve (ROC curve) and their respective 95% CI values. The validation analyses and tests were performed using RStudio software.
Data sources/ measurement	8*	For each variable of interest, give sources of data and details of methods of assessment (measurement). Describe comparability of assessment methods if there is more than one group	Pág. 5 – paragraph 4 and 5. Two logistic regression analyses were performed; the first considering as dependent variable people who considered their mental health condition as poor. The second regression analysis considering as dependent variable people who reported changes in relation to their mental health during the pandemic period (feeling more agitated, anxious, sad, irritated, discouraged, crying more easily, lonely, always thinking about COVID-19, work overload and/or sleeping difficulties, when compared to the pre-pandemic period).<br>Exploratory analysis was conducted for collinearity among the independent variables using the Variance Inflation Factor (VIF), and those with values greater than 10 were removed from statistical modeling. The modeling was performed using the backward

			stepwise selection method, in which one starts with a complete model (with all variables) and then removes the variables one by one and verifies the model's behavior. The best model considered was the one with the lowest Akaike Information Criterion (AIC) value. It is also worth mentioning that for the final model, the Odds Ratio (OR) with their respective 95% Confidence Intervals (95%CI) were calculated.
Bias	9	Describe any efforts to address potential sources of bias	Pág. 5 – paragraph 5 and 6. Exploratory analysis was conducted for collinearity among the independent variables using the Variance Inflation Factor (VIF), and those with values greater than 10 were removed from statistical modeling. The modeling was performed using the backward stepwise selection method, in which one starts with a complete model (with all variables) and then removes the variables one by one and verifies the model's behavior. The best model considered was the one with the lowest Akaike Information Criterion (AIC) value [20]. It is also worth mentioning that for the final model, the Odds Ratio (OR) with their respective 95% Confidence Intervals (95%CI) were calculated.<br>After exhausting all possibilities of analysis and choosing the final model (based on the criterion of lowest AIC value), the Hosmer-Lemeshow, likelihood ratio, CoxSnell, Nagelkerke, and McFadden tests were performed to validate the model. In addition, the predictive ability and accuracy of the models were checked based on the area under the Receiver Operating Characteristic curve (ROC curve) and their respective 95% CI values. The validation analyses and tests were performed using RStudio software.
Study size	10	Explain how the study size was arrived at	Pág. 5 – paragraph 1. Participants were invited to answer the questionnaire by accessing the link which was widely disseminated through the websites of the institutions participating in the research using e-mail, WhatsApp®, social networks (Facebook®, Instagram®, Twitter®) or blogs, in addition, people were invited from the network of contacts of the researchers involved. In addition, the participants were instructed to recruit other people from their social circle to participate in the survey, in order to obtain the expected sample.

Quantitative variables	11	Explain how quantitative variables were handled in the analyses. If applicable, describe which groupings were chosen and why	Pág. 5 – paragraph 2 and 3. After consistent analysis of the database, exploratory analyses were performed to characterize the profile of the people who answered the questionnaire.<br>To identify factors associated with the outcomes of interest, binary logistic regression was used based on the variables present on the "Social Thermometer" instrument (Questionnaire), which were dichotomized.
Statistical methods	12	(*a*) Describe all statistical methods, including those used to control for confounding	Pág. 5 – paragraph 4. Two logistic regression analyses were performed; the first considering as dependent variable people who considered their mental health condition as poor. The second regression analysis considering as dependent variable people who reported changes in relation to their mental health during the pandemic period (feeling more agitated, anxious, sad, irritated, discouraged, crying more easily, lonely, always thinking about COVID-19, work overload and/or sleeping difficulties, when compared to the pre-pandemic period).
		(*b*) Describe any methods used to examine subgroups and interactions	not applicable
		(*c*) Explain how missing data were addressed	not applicable
		(*d*) If applicable, describe analytical methods taking account of sampling strategy	not applicable
		(*e*) Describe any sensitivity analyses	not applicable
**Results**			
Participants	13*	(a) Report numbers of individuals at each stage of study—eg numbers potentially eligible, examined for eligibility, confirmed eligible, included in the study, completing follow-up, and analysed	not applicable
		(b) Give reasons for non-participation at each stage	not applicable
		(c) Consider use of a flow diagram	not applicable

Descriptive data	14*	(a) Give characteristics of study participants (eg demographic, clinical, social) and information on exposures and potential confounders	Pág. 6 – paragraph 3. A total of 2,698 people answered the questionnaire, with the majority of respondents being female (n=1,667, 61.7%), age range between 18 and 39 years (n=1,187, 44.0%), with 18 years as the minimum age and 89 years as the maximum, mean age 44 years, median 42 years, and standard deviation 15 years. The predominant race/color was white (n=1,463; 54.2%), married, widowed and separated or single (n=1,632; 60.5%), and with complete college education (n=1,989; 73.7%). The majority declared to be formal workers (n=1,109; 41.1 %), with income between 1 and 5 minimum wages (n=860; 31.9%) and declared not to receive any type of government assistance (n=2,438; 90.4%), as presented in Table 1.
		(b) Indicate number of participants with missing data for each variable of interest	Pág. 6. Table 1. Sociodemographic profile of the participants under study, Brazil, 2022 (N=2,698)
Outcome data	15*	Report numbers of outcome events or summary measures	Pág. 9 – paragraph 1,2,3,4 and 5. To validate the model presented in Table 2, it was verified that the accuracy capacity of the model through the area under the ROC curve showed a value of 0.83, besides the Hosmer-Lemeshow test (p=0.16), likelihood ratio (p=<0.01), CoxSnell (0.30), Nagelkerke (0.41) and McFadden (0.26).<br>In Table 2, it was possible to identify the factors associated with self-reported mental health change, and emotional change. It was found that people aged 18 to 39 years were less likely [OR: 0.37; 95%CI: 0.24 - 0.56] to consider their mental health status poorly compared to other age groups, as they were people who reported not feeling agitated or sad as a result of the isolation measures (OR: 0.33; 95%CI: 0.14 - 0.68).<br>Compared to the pre-pandemic period of COVID-19, people who reported not feeling more agitated, anxious, or tense have lower odds (OR: 0.53; 95%CI: 0.39 - 0.72) of considering their mental health status poorly compared to the post-pandemic period. The same occurs for people who reported not feeling more irritable (OR: 0.64; 95%CI: 0.46 - 0.88), more sad, discouraged or who cry more easily (OR: 0.35; 95%CI: 0.25 - 0.47) and with more difficulty sleeping (OR: 0.46; 95%CI: 0.34 - 0.64).

Also as protective factors, it was identified that people who did not start or increase the use of depressants or antidepressants because of the pandemic have fewer chances (OR: 0.15; 95%CI: 0.04 - 0.44) to consider their mental health status bad, as well as not being workers from risk groups for contamination by COVID-19 (OR: 0.67; 95%CI: 0.49 - 0.91).

Regarding risk factors, it was identified that people with complete higher education (OR: 1.97; 95%CI: 1.21 - 3.22) and people who reported using tranquilizers or antidepressants to cope with the current situation (OR: 2.04; 95%CI: 1.46 - 2.85) were more likely to consider their mental health status poorly.

Pág. 11 – paragraph 1 and 2. To validate the model presented in Table 3, it was verified that the accuracy capacity of the model through the area under the ROC curve presented a value of 0.78, besides the Hosmer-Lemeshow test (p=0.52), likelihood ratio (p=<0.01), CoxSnell (0.12), Nagelkerke (0.24) and McFadden (0.17).

In Table 3, it was possible to identify four protective and one risk variable. We identified that individuals who had elementary school (OR: 0.24; 95%CI: 0.10 - 0.58) or complete high school (OR: 0.30; 95%CI: 0.10 - 0. 60) were less likely to report changes in their mental health status in the pandemic period compared to the pre-pandemic period. Also, people who did not start or increase their use of depressants and/or antidepressants because of the pandemic (OR: 0.13; 95%CI: 0.03 - 0.53) and those who considered their mental health status poor (OR: 0.10; 95%CI: 0.04 - 0.18) were identified. In turn, people who reported increased consumption of ultra-processed foods (OR: 2.49; 95% CI: 1.43 - 4.61) were more likely to report changes regarding their mental health in the pandemic period compared to the pre-pandemic period.

Main results	16	(*a*) Give unadjusted estimates and, if applicable, confounder-adjusted estimates and their precision (eg, 95% confidence interval). Make clear which confounders were adjusted for and why they were included	Pág. 9 – paragraph 1,2,3,4 and 5. To validate the model presented in Table 2, it was verified that the accuracy capacity of the model through the area under the ROC curve showed a value of 0.83, besides the Hosmer-Lemeshow test (p=0.16), likelihood ratio (p=<0.01), CoxSnell (0.30), Nagelkerke (0.41) and McFadden (0.26).<br>In Table 2, it was possible to identify the factors associated with self-reported mental health change, and emotional change. It was found that people aged 18 to 39 years were less likely [OR: 0.37; 95%CI: 0.24 - 0.56] to consider their mental health status poorly compared to other age groups, as they were people who reported not feeling agitated or sad as a result of the isolation measures (OR: 0.33; 95%CI: 0.14 - 0.68).<br>Compared to the pre-pandemic period of COVID-19, people who reported not feeling more agitated, anxious, or tense have lower odds (OR: 0.53; 95%CI: 0.39 - 0.72) of considering their mental health status poorly compared to the post-pandemic period. The same occurs for people who reported not feeling more irritable (OR: 0.64; 95%CI: 0.46 - 0.88), more sad, discouraged or who cry more easily (OR: 0.35; 95%CI: 0.25 - 0.47) and with more difficulty sleeping (OR: 0.46; 95%CI: 0.34 - 0.64).<br>Also as protective factors, it was identified that people who did not start or increase the use of depressants or antidepressants because of the pandemic have fewer chances (OR: 0.15; 95%CI: 0.04 - 0.44) to consider their mental health status bad, as well as not being workers from risk groups for contamination by COVID-19 (OR: 0.67; 95%CI: 0.49 - 0.91).<br>Regarding risk factors, it was identified that people with complete higher education (OR: 1.97; 95%CI: 1.21 - 3.22) and people who reported using tranquilizers or antidepressants to cope with the current situation (OR: 2.04; 95%CI: 1.46 - 2.85) were more likely to consider their mental health status poorly.<br>Pág. 11 – paragraph 1 and 2. To validate the model presented in Table 3, it was verified that the accuracy capacity of the model through the area under the ROC curve presented

			a value of 0.78, besides the Hosmer-Lemeshow test (p=0.52), likelihood ratio (p=<0.01), CoxSnell (0.12), Nagelkerke (0.24) and McFadden (0.17).<br>In Table 3, it was possible to identify four protective and one risk variable. We identified that individuals who had elementary school (OR: 0.24; 95%CI: 0.10 - 0.58) or complete high school (OR: 0.30; 95%CI: 0.10 - 0. 60) were less likely to report changes in their mental health status in the pandemic period compared to the pre-pandemic period. Also, people who did not start or increase their use of depressants and/or antidepressants because of the pandemic (OR: 0.13; 95%CI: 0.03 - 0.53) and those who considered their mental health status poor (OR: 0.10; 95%CI: 0.04 - 0.18) were identified. In turn, people who reported increased consumption of ultra-processed foods (OR: 2.49; 95% CI: 1.43 - 4.61) were more likely to report changes regarding their mental health in the pandemic period compared to the pre-pandemic period.
		(*b*) Report category boundaries when continuous variables were categorized	not applicable
		(*c*) If relevant, consider translating estimates of relative risk into absolute risk for a meaningful time period	not applicable
Other analyses	17	Report other analyses done—eg analyses of subgroups and interactions, and sensitivity analyses	not applicable
**Discussion**			
Key results	18	Summarise key results with reference to study objectives	Pág. 12 – paragraph 1 and 2. The present study aimed at the perception of changes in mental health, emotional problems and their determinants in the midst of the covid-19 pandemic in Brazil. We observed, more prevalence of situations such as being more agitated, anxious, sad, irritated, discouraged, crying more easily, and being more lonely. Part of the subjects also reported more work overload and/or sleeping difficulties.

			We also verified that among the resources to mitigate emotional problems and relief anxiety was to resort to self-medication and alcohol use. We identified that people with complete college education (OR: 1.97; 95%CI: 1.21 - 3.22) and people who reported using tranquilizers or antidepressants (OR: 2.04; 95%CI: 1.46 - 2.85) self-reported better health status. Income was a marker for people's access to these resources, which somewhat highlights disparities among the groups studied.
Limitations	19	Discuss limitations of the study, taking into account sources of potential bias or imprecision. Discuss both direction and magnitude of any potential bias	Pág. 17 – paragraph 1. As limitations of the study, we highlight the study design employed. Since this was an online survey, several social segments were not included (such as people in situations of social vulnerability). Barrier of access to the Internet may have caused the involuntary exclusion of these groups and influenced the estimates of the proportion of responses, justifying the difference between the profiles of the study sample compared to the Brazilian population. The analysis of self-reported conditions rather than formal mental health diagnoses is a limitation to be considered in this work. Thus, it is important that further studies be conducted to understand the real impact that the COVID-19 pandemic may have caused on the mental health of the Brazilian population.
Interpretation	20	Give a cautious overall interpretation of results considering objectives, limitations, multiplicity of analyses, results from similar studies, and other relevant evidence	Pág. 14 – paragraph 3.When discussing the relationship of individuals with their residential environment in a physically distant situation in Brazil during the pandemic, one study signaled that physical distancing with prolonged stay inside the home was a source of stress for most participants in the country. The authors believe that such stress occurs mainly due to mobility restrictions, intensification of family interaction caused by the situation of being confined for several months.
Generalisability	21	Discuss the generalisability (external validity) of the study results	Pág. 14 – paragraph 4. A longitudinal study that assessed the impact of lockdown on mental health during the first year of the COVID-19 pandemic in a general sample of the French population identified that such a health crisis strongly affected the occurrence and persistence of depression, anxiety, and post-traumatic stress disorder over time . The prevalence of depression symptoms among adults in the United States increased dramatically during the early months of the COVID-19 pandemic. A study

			that sought to understand the impact of the pandemic on people with a history of depression showed that this relationship increased the chances of negative effects of the pandemic on various aspects of life.
**Other information**			
Funding	22	Give the source of funding and the role of the funders for the present study and, if applicable, for the original study on which the present article is based	not applicable

*Give information separately for exposed and unexposed groups.

Note: An Explanation and Elaboration article discusses each checklist item and gives methodological background and published examples of transparent reporting. The STROBE checklist is best used in conjunction with this article (freely available on the Web sites of PLoS Medicine at http://www.plosmedicine.org/, Annals of Internal Medicine at http://www.annals.org/, and Epidemiology at http://www.epidem.com/). Information on the STROBE Initiative is available at www.strobe-statement.org.
